# Danaher’s Ethical Behaviourism: An Adequate Guide to Assessing the Moral Status of a Robot?

**DOI:** 10.1007/s11948-020-00230-4

**Published:** 2020-06-16

**Authors:** Jilles Smids

**Affiliations:** 1 Philosophy & Ethics, Eindhoven University of Technology, Eindhoven, The Netherlands; 2 Medical Ethics, Philosophy and History of Medicine, Erasmus MC, University Medical Centre Rotterdam, Rotterdam, The Netherlands

**Keywords:** Robot, Moral status, Ethical behaviourism, Inference to the best explanation, Robot ethics

## Abstract

This paper critically assesses John Danaher’s ‘ethical behaviourism’, a theory on how the moral status of robots should be determined. The basic idea of this theory is that a robot’s moral status is determined decisively on the basis of its observable behaviour. If it behaves sufficiently similar to some entity that has moral status, such as a human or an animal, then we should ascribe the same moral status to the robot as we do to this human or animal. The paper argues against ethical behaviourism by making four main points. First, it is argued that the strongest version of ethical behaviourism understands the theory as relying on inferences to the best explanation when inferring moral status. Second, as a consequence, ethical behaviourism cannot stick with merely looking at the robot’s behaviour, while remaining neutral with regard to the difficult question of which property grounds moral status. Third, not only behavioural evidence ought to play a role in inferring a robot’s moral status, but knowledge of the design process of the robot and of its designer’s intention ought to be taken into account as well. Fourth, knowledge of a robot’s ontology and how that relates to human biology often is epistemically relevant for inferring moral status as well. The paper closes with some concluding observations.

## Introduction

The debate about the moral status of robots is gaining more and more traction.^1^ For example, would we wrong a very sophisticated and apparently intelligent robot by cutting of its electrical power supply (Cf. Sparrow 2004)? It is generally acknowledged that much uncertainty surrounds the issue of determining a robot’s moral status (Coeckelbergh 2014; Gunkel 2018; Schwitzgebel and Garza 2015). Recently, John Danaher has given a bold, extensive, and interesting defence of a distinctive theory on how this moral status should be determined: ‘ethical behaviourism’ (Danaher 2019). The basic idea of this theory is that a robot’s moral status is determined solely, or at least decisively, on the basis of its observable behaviour. If it behaves sufficiently similar to some entity that has moral status, such as a human or an animal, then we should ascribe the same moral status to the robot as we do to this human or animal. Knowing what goes on “on the inside” does not matter (sec. “Defending Premise (1)”). If sound, this theory would do a lot to reduce the mentioned uncertainty by yielding determinate answers to questions of robotic moral status. In this paper, I will critically assess Danaher’s ethical behaviourism, and argue that, despite Danaher’s impressive defence, it fails because of its exclusion of any other type of evidence beyond mere behavioural performance.

It is important to get robots’ moral status right, since if we fail to recognize robots’ moral standing, we might abuse them or treat them in other ways that are morally wrong (Neely 2014). But we can err on both sides: there are also costs to ascribing too much moral status to robots. Children and adults alike may invest too much time and energy in caring for a robot whose moral status doesn’t warrant such care. These resources could have been spent to worthier causes: fellow humans (Bryson 2010, 2018). This could go as far as risking one’s life to save a robot that one mistakenly believes to be worth it (Schwitzgebel and Garza 2015). On the level of policymaking, there also is the cost of misspending resources. In addition, having a realistic view of reality is inherently worthwhile. The consequence of ethical behaviourism (EB) is that robots should be welcomed into our moral circle. Or, at least those robots that have sufficient moral standing, which according to Danaher will be among us in the near future, if not already. For, i follows from EB that the threshold for moral status may not be that high. Given this potentially rather far-reaching consequences, it is worth undertaking a critical investigation into the merits of EB.

I will start with laying out the basic structure of EB (Sect. "Ethical Behaviourism: An Overview of the Theory"). I then argue that an interpretation of EB as relying on an argument from analogy faces serious difficulties. Instead, EB is most plausibly construed as relying on abductive reasoning, or, inferences to the best explanation (Sect. "Ethical Behaviourism Relies on Abductive Reasoning"). However, if that is right, then it follows that EB in fact seeks to infer to the presence of some ontological or metaphysical property that grounds moral status, and thus, that EB cannot remain neutral with regard to the difficult question of which property is the best candidate for grounding moral status (Sect. "What Goes on ‘on the Inside’ *Does* Matter"). I subsequently argue that not only behavioural evidence ought to function as the explanans of an inference to the best explanation, but knowledge of the design process of the robot and of its designer’s intention ought to be allowed as well (Sect. "The Relevance of the Design Process of Robots"). In addition, also knowledge of a robot’s ontology and how that relates to human biology is often epistemically relevant (Sect. "Ontology Matters (a Lot)"). In Sect. "Conclusing remarks", I make some concluding observations and sum up the positive and more constructive results from my analysis of EB: I give some recommendations for how to go about in assessing a robot’s moral status and for further research.^2^

## Ethical Behaviourism: An Overview of the Theory

Danaher gives the following succinct statement of his EB:

If a robot is roughly performatively equivalent to another entity whom, it is widely agreed, has significant moral status, then it is right and proper to afford the robot that same status (Danaher 2019, p. ‘The Sophia Controversy’).^3^

Danaher subsequently clarifies two terms that are central to his EB. First, an entity’s having ‘moral status’ means that it is not completely up to us how we treat that entity; there are ethical restrictions to our behaviour toward that entity. Second, ‘rough performative equivalence’ (RPE) means ‘consistently behav[ing] like’ the other entity with which the robot is compared. The modifier ‘rough’ is justified on the basis of the fact that entities with the same moral status, like us humans, never perform exactly the same set of behaviours. Here, behaviour should be understood rather broadly as including all ‘external observable patterns’, like moving my legs, uttering sounds and the like. But, notably, it also encompasses brain activity, which is observable by means of sophisticated scanning techniques.^4^

The fundamental idea underlying EB and the main reason to accept the theory is the fact, as Danaher sees things, that when it comes to determining some entity’s moral status, its observable behaviour is our only source of knowledge. In our daily practice, we continuously infer the moral status of others, such as humans and animals, from their behaviour. We are epistemically limited beings who lack direct access to whatever metaphysical properties ground these others’ moral status. Instead, we necessarily must rely on our observations. It is precisely because EB respects these epistemic limits that the theory is to be favoured as the approach to assessing moral status. Danaher summarizes his argument for the view that we should grant a robot the same status as some other entity to which the robot shows rough performative equivalence as follows:

[T]he reason why one should accept ethical behaviourism is that it is an essential feature of day-to-day ethical practice: inferences from behaviour are the *primary and most important* source of knowledge about the moral status of others; if we did not rely on these inferences, the identification and protection of moral status would be impractical. (sec. “Defending Premise (1)”, italics JS).

This might read as merely describing how we go about in ascribing moral status to others, but Danaher hastens himself to emphasize that EB is a “normative and meta-empirical thesis” (ibid.), that prescribes two things to us. First, it states that externally observable behaviour is the primary and most important, or, as Danaher writes at other places (ibid.), the only^5^ evidence on which we ought to base our ascriptions of moral status to others (humans, animals, robots, etc.). Second, it tells us to interpret that evidence according to what Danaher calls “The Comparative Principle of EB”:

If an entity X displays or exhibits roughly equivalent behavioural patterns (P_1_…P_n_) to entity Y, and if it is believed that those patterns ground or justify our ascription of rights and duties to entity Y, then either (a) the same rights and duties must be ascribed to X or (b) the use of P_1_…P_n_ to ground our ethical duties to Y must be reevaluated (ibid.).

This means, using an example of Danaher, that we owe a robot that exhibits roughly similar (and thus not very sophisticated) behavioural patterns as mice or chicken the same moral respect that is owed to mice and chicken. Most people think that we owe it to these animals that we care for their welfare and not harm them without sufficient reason. According to EB, we owe this to the robot as well (sec. “Defending Premise (2)”).

Danaher describes his ethical behaviourism as the “application of methodological behaviourism… to the ethical domain” (sec. “Defending Premise (1)”). Methodological behaviourism is the normative view that not what goes on in the inside (mental states like beliefs, desires, etc.) is psychology’s proper object of study, but externally and publicly observable behaviour (Graham 2019). EB applies this methodological injunction to the assessment of the moral status of others. According to EB, the observable behaviour of some entity should be sufficient ground or warrant for ascribing moral status to that entity. Note that the comparative principle of EB, cited in full above, provides the more detailed procedure of how an entity’s moral status can be inferred from its patterns of behaviour.

Just like methodological behaviourism does not deny the existence of inner mental states, EB does not need to deny that humans or other entities have inner mental states. Moreover, it is also compatible with the view that these inner mental states and especially their related metaphysical properties, such as consciousness or sentience, are the ultimate grounds for the entities having moral status. In that sense, EB is consistent with acknowledging the relevance of ontology. However, EB stresses that manifest behaviour is our only, or our sufficient, or our primary evidence for ascertaining that an entity has such metaphysical properties. Hence, the ultimate epistemic ground for ascribing moral status is mere behaviour. In this way, EB explicitly aims to side-step the debate on the ontological grounds of moral status.

Having laid out the basics of EB, it is instructive to consider how EB relates to classical psychological behaviourism, a view which includes commitment to methodological behaviourism. Briefly, psychological behaviourists like JB Watson and BF Skinner claimed that behaviour should be explained without recourse to inner mental states and psychological processes. Instead, psychology should seek to establish law-like relationships between external stimuli and resulting externally observable behaviour. However, psychological behaviourism faces a fundamental problem, viz. the impossibility to give satisfactory explanations of behaviour without postulating inner mental events and processes. Accordingly, behaviourism has lost much of its plausibility (Graham 2019; Heil 2004)**.**^6^ As I will argue below, the most fundamental problem of ethical behaviourism is rather similar. That is, when it comes to assessing a robot’s moral status, EB strongly denies the epistemic relevance of anything else beyond externally observable robotic behaviour.

A second issue worth consideration concerns the relation between the core ideas of EB and our tendency to display anthropomorphizing responses to robots. That is, we tend to attribute humanlike characteristics to robots in order to make sense of their behaviour. So, we often talk about robots, especially social robots as having emotions, beliefs, desires, and the like in order to interpret their outward appearance and actions (Darling 2016; Duffy 2003; Nyholm 2020; Turkle et al. 2006)**.** If we would also believe that robots in fact possess those characteristics that we attribute when anthropomorphizing, then consistency would seem to require to also ascribe moral status. For, if we really believe that robots have intentions, can think, feel emotions, then we ought to grant them the moral status that we consider to be bound up with experiencing such mental states. However, when explicitly asked, most people are well aware that the robots such as Aibo and Paro do not really have the emotions, intentions and beliefs they project to them (Turkle et al. 2006)**.** As Turkle explains, it is because robots “push our Darwinian buttons” that we ascribe mental states to robots that we at the same time believe they do not really have. So, when a robot makes eye contact and traces our movements, we automatically respond to it as if it were a social being, even shortly after being explained the detailed mechanisms that enable the robot to do so (Turkle 2007, pp 3–4). In other words, when a robot exhibits characteristic social cues, our biological make-up causes us to unreflectively respond accordingly, in characteristic social manners.

People thus usually are very well able to distinguish between the dissimilar internal states that, respectively, enable robots and humans to behave in social manners. Nevertheless, ethical behaviourism instructs us to exclusively look at the behavioural artifice. In that respect, is requires us to abstract away from considerations that we in fact have when thinking about robots and their interactions with us. Of course, as an explicitly normative theory, EB legitimately can do that. But contrary to Danaher’s claim, in this respect, EB does not match daily practice. The literature on anthropomorphizing cited above indicates that we rely on more than inferences from observable behaviour alone. What we know from the design and inner workings of robots is also important for us when thinking about robotic moral status.

It follows that we cannot interpret EB as an approach to ascribing moral status that would match nicely with the way we act when we anthropomorphize robots. If EB were correct, then we ought to show our typical anthropomorphising nurturing responses to any robot dog that is roughly performatively equivalent to a real dog. Or at least, we should treat it with similar consideration, even if in the case of a robot dog the appropriate ways of expressing that consideration would be different. However, most of us think there is a gap between how we in fact react to robots, and the reactions merited by their moral status as perceived by us.^7^ I conclude that EB’s insistence to disregard the design and inner processes of robots is implausible. Despite that drawback, the theory could still be true or plausible on other grounds, so I will now analyse the theory in more detail.

## Ethical Behaviourism Relies on Abductive Reasoning

The strongest version of ethical behaviourism crucially relies on abductive reasoning, or inferences to the best explanation. To see the need for interpreting EB along such lines, it is instructive to first look at the problems that arise if we, alternatively, construe EB as involving analogical arguments. At face value, Danaher might seem to classify his EB as relying on such arguments:

The argument works off a principle of analogy: if case A is like case B (in all important respects) then they should be treated alike. So, for example, if animals are owed certain moral duties—e.g. not to be mistreated or subjected to needless cruelty—and if robots are roughly performatively equivalent to animals, then, following this argument, robots are owed equivalent duties. (sec. “The Sophia Controversy”)^8^

So, the idea would be that we observe the behavioural patterns of, for example, a chicken, and note that the behavioural pattern of a certain robot is roughly equivalent. We know that we owe chicken some moral consideration, and, by way of further analogy, we infer that the robot is also similar to the chicken in this respect. Thus, we owe the robot a similar moral consideration.

However, so construed, EB would have two insurmountable problems. First, an analogical argument only confers some degree of support to its conclusion (Bartha 2019). The degree of support may vary depending on various considerations, but the above analogical argument at most delivers moderate support for robotic moral status, nothing of the sort EB intends to deliver. For, nowhere in his statement of how EB works does Danaher add qualifiers to EBs conclusions, such as that robots are possibly, or likely owed equivalent duties. Instead, there seems to be full symmetry between the confidence with which we ascribe moral status to the comparator case and the confidence with which we ought to ascribe the same moral status to the robot in question.

Second, if EB would rely on analogical arguments, it would be unable to account for the moral status of robots that have no other being to which their behaviour is sufficiently analogous. Suppose that, applying EB, we ascribe moral status to some robot dog, based on its rough performative equivalence to some biological dog. Now, imagine the following robot animal that has the same AI-based processing capacities as the robot dog. Its behaviour is designed to be a mixture of a number of wildly different animals: it finds food like a hamster, if it is hit, it roars like a lion, if you say kind words to it, it wags its tail like a dog and starts spinning like a cat. And so on. After all, that is what robot designers can do, would they wish so. Clearly, our somewhat undetermined robot animal will lack rough performative equivalence with any plausible comparator animal. However, if robot dogs have moral status, then this robot animal does as well. Whatever metaphysical properties may ground moral status, if a robot dog has them, then our robot-animal will have them as well. If the robot dog’s manifest pain behaviour grounds our belief that it is sentient and deserves moral consideration, then our robot animal will as well, based on its lionlike pain behaviour. The same goes for “having sufficiently sophisticated cognitive capacities”, and any other potential moral status grounding property.

From this analysis of the robot animal we can generalize: the same properties that may ultimately ground moral status, such as sentience and cognitive capacities, are compatible with widely different behavioural patterns. If we are merely looking for analogous behavioural patterns to assess moral status, then for some sets of behavioural patterns we will fail to find a comparator case, and consequently fail to ascribe moral status to the being that exhibits that pattern.

However, the fact that we feel confident in ascribing, for example, sentience to animals as dis-analogous to us humans as hamsters, lions, dogs, and cats, suggests that we do not rely on analogical reasoning, but instead on abductive reasoning: we infer from their different behavioural patterns to the best explanation available, viz. that all these animals are sentient (Cf. Graham 1998, pp 51–63). Assuming that sentience is a property sufficient to ground some moral status, it follows that all these animals share in that status. Thus, applying abductive reasoning, we can avoid what Graham calls the problem of ‘parochialism’ (op cit., p52), i.e., our inability to ascribe minds, and thus moral status to beings dissimilar to us humans.

At this point, it is instructive to consider how the problem of determining a being’s moral status is related to solving the problem of whether that being has a mind (and what kind of mind) and thus to the classic problem of other minds. This is the problem of how to justify our beliefs that others also have feelings, thoughts, etc., in short, that they have a mind.^9^ Many of the properties that are proposed as grounds for having moral status entail having a mind.^10^ Danaher mentions several (sec. “Defending Premise (1)”), such as personhood and having interests, and explicitly discusses having (sophisticated) cognitive capacities, and sentience and consciousness as being potential ultimate grounds for having moral status. Vice versa, having a mind or mental life seems to at least involve sentience, which is often thought to ground at least some moral status.

An inference to the best explanation is widely viewed as the strongest solution available to the problem of other minds (Avramides 2019; Chalmers 1996; Graham 1998; Pargetter 1984). The best explanation for the multiple behaviours of other humans seems that they have a (rational) mind of their own. For example, consider a mother that gives her child fruits to eat, tells her child that she wants it to grow up healthily, etc. The best explanation for this set of behaviours is to hold that the mother has a mind, loves her child, and in fact believes that eating fruits helps to grow up in health. Therefore, we are justified in believing that the mother has a mind like us.

The strongest version of EB similarly relies on abductive reasoning. Let us suppose for purposes of discussion that sentience is an ontological property that confers a being significant moral status.^11^ Suppose further that we see a dog being hit hard and responding with typical pain behaviour such as screaming, grimacing, and shrinking away. The best explanation for the fact that dogs when hit hard scream, grimace, shrink away, and next time avoids the cruel human that hit them, is the view that they have the capacity to feel pain and thus are sentient. Consequently, we know that the dog has some moral standing, since we view being able to suffer as conferring moral status. Now, what EB tells us to do for a robot dog that displays roughly the same behaviour under the same circumstances, is to apply the same abductive reasoning and infer that the robot dog has similar moral status. Thus, we might say that the analogy between our approach to the biological dog and the robot dog lies in how we infer to sentience as the best explanation of apparent pain behaviour.^12^

Note how, in the first place, we also base our ascription of moral status to the biological dog on an inference to the best explanation.^13^ Our knowledge of our own pain experiences serves to ‘state the hypothesis’ (Cf. Pargetter 1984, pp 158–9, 162). Thus, I know from my own case that when I am in pain, I behave in certain characteristic ways and have a qualitatively distinct experience that we call ‘being in pain’. Then the hypothesis that best explains the pain behaviour I observe in a dog is that that dog experiences qualitatively sufficiently similar pain sensations. This is the case even though doglike pain behaviour cannot be said to be roughly performatively equivalent to my own pain behaviour. Yet, I infer that dogs are sentient like me, or at least that I am justified in believing that dogs are sentient, even if most of the time I am not engaged in explicit reasoning about whether or not the dog can experience pain and is sentient.^14^

My explicit interpretation of EB as crucially relying on inferences to the best explanation seems clearly consistent with Danaher’s presentation of EB. Danaher emphasizes that EB is compatible with different views as to which metaphysical property is the ultimate ground for moral status. EB can adapt to any of these by “arguing that a sufficient epistemic warrant for believing in the existence of this metaphysical property can be derived from an entity’s observable behavioural patterns” (sec. “Defending Premise (1)”). Thus, the existence of the relevant metaphysical property is inferred from observable behaviour. This is explicit from a passage a little bit further in the paper:

The ethical behaviourist points out that our ability to *ascertain* the existence of each and every one of these metaphysical properties is ultimately dependent on some inference from a set of behavioural representations. Behaviour is then, for practical purposes, the only insight we have into the metaphysical grounding for moral status (sec. “Defending Premise (1)”, Italics JS).

It is hard to see what type of reasoning other than abduction can conclude to the presence of some metaphysical property from such qualitatively different ‘data’ as observable behavioural patterns. However, once we recognize EB’s reliance on this type of reasoning, it becomes clear that contrary to Danaher’s claim, “what’s going on ‘on the inside’” (sec. “The Sophia Controversy”) *does* matter, not merely ethically, but also epistemically.

## What Goes on ‘on the Inside’ *Does* Matter

From the characterization of EB given in the previous section, it follows that EB cannot remain neutral, or “strictly agnostic” (sec. “Defending Premise (2)”, Italics original) with respect to which metaphysical properties ultimately ground moral status. The reason is that it is not ‘having moral status’ that serves as the hypothesis that explains observable behaviour, but rather one of the various metaphysical properties that may ground moral status, such as sentience or having cognitive capacities. If we ask why an animal exhibits typical pain behaviour, it will not help to answer that it has moral status. Instead, we are confident that the animal is a sentient being, because sentience causes pain behaviour. Subsequently, we can infer its moral status from its being sentient. Therefore, unlike what the Comparative Principle seems to suggest, our belief that certain behavioural “patterns ground or justify our ascription of rights and duties” to some entity, is not based on a direct inference from behaviour to moral status. For being justified in ascribing moral status, the intermediate inference from behaviour to the relevant metaphysical property is essential.

Danaher seems to recognize this when discussing how we could assess whether some “behaviourally-sophisticated robot” is roughly “performatively equivalent to a competent adult” (sec. “Defending Premise (2)”). He claims that sweating and probably also characteristics such as having two legs and a humanlike skin, are not necessary for moral status. However, if we would directly infer from behaviour to moral status, then it is hard to see why sweating is any less relevant than being sentient. Without specifying the metaphysical principle that grounds having moral status, we cannot determine which behaviours are relevant to assessing moral status and which not.

Such a narrow selection of behaviour that is relevant for moral status may appear an astonishing move, given Danaher’s emphasis on rough performative equivalence and sentences like “If a robot looks and acts like a being to whom moral status is afforded then it should be afforded the same moral status” in his conclusion. But if we recognize EB’s abductive reasoning, it makes perfect sense. Only that subset of robotic behaviour is relevant that can best be explained by inferring the presence of the relevant moral status grounding property that we are looking for. And indeed, Danaher suggests that our method to asses performative equivalence to adults may be close to, or coincide with the Turing test “on the grounds that it is cognitive behaviour that really matters when it comes to moral status” (sec. “Defending Premise (2)”). Thus, only cognitive behaviour matters, and it is singled out on the basis of the chosen property that grounds moral status, viz. sophisticated cognitive capacities.

At this point, it is important to note the following. Referring to cognitive behaviour instead of sophisticated cognitive capacities (sec. “Defending Premise (2)”) is of no help here. For, the only reason to single out cognitive behaviour as decisive for moral status ascription is the assumption that sophisticated cognitive capacities ground moral status. The lesson is general: EB cannot maintain neutrality with respect to the question which property ultimately grounds moral status. This is an unfortunate consequence, since it follows that for putting EB to work, one has to adopt a specific philosophical view regarding which metaphysical property ground moral status. As is evident from the fact that various discussions in the literature single out different metaphysical properties, this is an important source of epistemic and moral uncertainty regarding robotic moral status. For example, Neely (2014) bases her discussion on “having interests”, Sebo (2018) (only touching on the issue of robotic status) focuses on “sentience”, and Agar (2019) on “having a mind”.^15^

## The Relevance of the Design Process of Robots

Once we adopt abductive reasoning to asses robotic moral status, the question arises why only observable behaviour would be permitted in the explanandum. Or, somewhat less strict, why ought observable behaviour to be decisive. To put it differently, does methodological behaviourism still make sense? Once we recognize that we look at the robot’s behaviour with the aim to infer whether or not the robot has the metaphysical property that we believe grounds moral status, why not allow all evidence that might be of relevance to making a justified inference?

To address this question, consider a very sophisticated robot dog. It walks in characteristic doglike manner, it starts to wag its tail when it sees its owner, if it is being kicked it screams very realistically, looks as if in pain, flees, and so on. So, let us just stipulate that it is roughly performatively equivalent to a real dog. However, despite its behavioural realism, it is still recognizable as a robot dog.

A comparison between the robot dog and our biological dog referred to earlier above will show that when it comes to ascribing moral status to robots, it is epistemically irresponsible to grant behavioural performances the role of decisive evidence. Let us suppose again that sentience is an ontological property that confers a being significant moral status. We see a dog being hit hard and responding with typical pain behaviour such as screaming, grimacing, and shrinking away. The best explanation for the fact that dogs when hit hard scream, grimace, shrink away, and next time avoids the cruel human that hit them, is the view that they have the capacity to feel pain and thus are sentient. Consequently, we know that the dog has some moral standing.

In case of the robot dog, however, this inference to the robot dog experiencing pain as the best explanation for its externally observable ‘pain’ behaviour, breaks down. When we ask what best explains the dog’s pain behaviour, we now have an excellent alternative explanation. Compare:

- The robot dog screams, grimaces, shrinks away, etc. because it feels pain.
- The robot dog screams, grimaces, shrinks away, etc. because it is designed to simulate these behaviours when hit hard.

Clearly, (b) is by far the most plausible explanation. The dog is designed to display pain behaviour, but not to in fact experience pain. The artificial nature of the dog separates what is intimately connected in real dogs: having inner pain sensations and exhibiting pain behaviour. It is characteristic (and very fortunate!) for robotic design that it is possible, at least up to a certain extent, to realize behavioural equivalence without having to design a mental life equivalent to that of a real dog. However, for that very reason, behavioural equivalence does not unconditionally support an inference to the presence of mental life.

We see that in case of the robot dog, behavioural evidence is not decisive. Our knowledge of its designers’ intentions (its efficient cause) leads us to a conclusion that is the opposite of where the behavioural evidence points to, if it has any weight at all. For, we currently have no idea how it would even be possible to design a robot dog that can feel pain. And even if we knew, it would complicate the design of the robot for no additional gains with regard to its (outward) functionality. So, what we know from the design process of the robot dog makes it nearly certain that the robot dog is not sentient. Therefore, insofar as we hold that sentience grounds moral status, we have very little reason to think that the robot dog has moral status.

Danaher discusses what he calls the “Different Efficient Cause Objection”, responding to Hauskeller (2017). Hauskeller investigates the question whether we would be missing anything from a robot lover designed to behave exactly as a real lover. The passage from Hauskeller that is quoted by Danaher (sec. “Defending Premise (1)”), was inspiration for my robot dog case, so let me rephrase it to make explicit the abductive reasoning involved^16^:

We have a machine that behaves exactly as if it were self-aware. Now we have two possible explanations:

- It really is self-aware.
- It has been designed and programmed to behave exactly as if it were self-aware.

Since in this case, assuming that it is possible to design such robot, explanation (d) clearly is more plausible, and therefore, we have no basis to infer that the robot lover is self-ware.

In response, Danaher argues against the view that “any entity that is designed and manufactured cannot have significant moral status” (sec. “Defending Premise (1)”). However, those arguing for the relevance of the design process are not at all committed to that view. Hauskeller, for example, makes explicit that he does not want to categorically exclude the possibility of future robotic real persons (op cit., p 203), which I think he would probably grant moral status. The point of the above argument is much more modest: in this specific case, we have no reason to ascribe moral status, because we have no reason to infer that the robot is self-aware. Again, in this case, robotic behaviour does not justify inference to self-awareness, because we have an alternative explanation: it is designed to behave as if it is self-aware, and nothing of what we further know about the design process gives us reason to think that the apparently self-aware behaviour is also caused by the machine being in fact self-aware. It is possible to resist this conclusion, by arguing that it is impossible, or at least very hard, to create a machine that behaves exactly as if it is self-aware, without being self-aware. Since such a robot is still futurity, this response might possibly turn out to be correct. But given our strong natural tendency to respond socially to robots that exhibit social cues, it seems not too difficult to build robots that behave exactly as if it was self-aware. It just has to exhibit the behaviour that leads humans to ascribe intentions, beliefs, emotions, and the like, all of which imply self-awareness. Similar reasoning applies to the robot dog apparently in pain.

To conclude**,** both examples show that in addition to behavioural evidence, knowledge of the design process of a robot and of its designer’s intentions can be highly relevant to finding out whether we can infer moral status from robotic behaviour. In the next section, I will argue that the same is true for knowledge of a robot’s ontology.

## Ontology Matters (a Lot)

As part of his meticulous defence of EB against potential criticisms, Danaher discusses the objection that knowledge of the ontology of robots is essential to assessing their moral status. However, he discusses only one way in which ontology could matter for moral status, namely, that “being made of the right stuff would be necessary for moral status” (sec. “Defending Premise (1)”). He seems to construe the objection as the idea that in order to have moral status, an entity needs to have a definite ontology, such as a human biology. So, the idea would be that, for example, only members of the human species would have moral status, just by virtue of their biology. However, there is a different way in which ontology can matter for moral status.

Knowledge of a robot’s ontology can give us insight into what metaphysical properties it may or may not have. Thus, unlike Danaher’s discussion seems to assume, ontology does not matter as such, but for epistemic purposes: ontology is relevant in assessing whether a robot might have a certain metaphysical property. Consider the robot dog again. We know that it lacks the nerves, a central nervous system, and all other relevant biology that enable biological dogs to feel pain. The biological dog, however, shares with us a common evolutionary ancestry, which made the brains of dogs and humans sufficiently similar for us to infer that dogs feel pain. We know that when hit, certain physiological processes culminate in pain sensations. Our biological continuity to a dog supports our abduction that a screaming dog feels pain in ways that have some similarity to our human pain experiences. Al these considerations lack in case of the robot dog. Of course, it cannot be categorically excluded that a robot dog could be designed in a way that enables it to have pain sensations. However, nothing from what we know about the robot dog makes this plausible.

Thus, when asking for both a biological dog and a robot dog whether each is sentient, our epistemic situation differs profoundly. Therefore, when we apply this additional knowledge in asking what explains the robot dog’s behaviour, the initial likelihood of (a) in the choice between (a) and (b) becomes significantly lower:

- The robot dog screams, grimaces, shrinks away, etc. because it feels pain.
- The robot dog screams, grimaces, shrinks away, etc. because it is designed to simulate these behaviours when hit hard.

Accordingly, taking ontology into account, we have even more reason to favour (b) over (a) than we already have based on knowledge of the design process. And so, we have even more reason to conclude that, for all of we know, the robot dog very likely is not sentient.

Sebo (2018) has an instructive comparison between a lobster and a robot lobster that makes the same point. He argues that while we are uncertain in both cases, we are less confident that the robot lobster is sentient than that a ‘functionally identical’ robot lobster is sentient. His crucial appeal also is to ontology: unlike the robot lobster, the biological lobster shares our evolutionary ancestry, and is physiologically continuous to us.^17^

Let me give another illustration of the epistemic relevance of robotic ontology for moral status ascription. Warwick (2012) gives a fascinating discussion of his research into robots with biological brains. Warwick builds a biological brain by placing neurons from a rat foetus into a suitable medium. The neurons then spontaneously grow and develop a neural network that exhibits electrical activity, even without stimulation. When this network is connected to a robot, external signals are transferred and turn on the robot motor. Warwick speculates about the possibility that a similar biological neural network could acquire consciousness. Specifically, would the same number of neurons as contained in a human brain, 100 billion, organized in 3-D network structure be conscious?

What interests me here is how Warwick argues for the possibly of consciousness, a property relevant to moral status. He seems to argue that biological similarity points to possible similar functionality, such as consciousness. To be clear, he is not at all engaged in speciesism. Rather, from the biological make-up of such artificial biological brain, he infers that it may very well be conscious. Of course, a silicon ‘brain’ may ground consciousness as well. But so far, we do not *know* that it can, whereas for biological brains, we do. So, here again we see that knowledge of robotic ontology may matter for assessing moral status. More generally, many authors discussing robotic moral status adhere to the idea that having a human-like biology is an additional epistemic ground for thinking that a robot may be sentient (Cf. Agar 2019; Harris 2019; Nyholm 2020; Sebo 2018).

## Concluding remarks

We have seen that when it comes to assessing robotic moral status, we have more sources of relevant evidence than mere behavioural performance. Knowledge of the design process and the robot’s ontology are highly relevant as well. This undercuts each of the three strands that can be identified in Danaher’s justification for his ethical behaviourism (all in sec. “Defending Premise (1)”). First, EB would square with how we in fact go about in ascribing moral status. It is said to be practical to ascertain moral status in this way, and the fact that it works well would confer some initial plausibility to EB. However, as we have seen, we do not rely on mere behavioural evidence, but also take into account our knowledge of robotic ontology and design process. This also directly falsifies Danaher’s claim, second, that we have no other way than doing it in EBs way. When we rely on abductive reasoning and allow all relevant evidence, our method is no longer a form of ethical behaviourism. Third, the idea that EB has the virtue of “respecting our epistemic limits” because we lack direct access to the metaphysical properties that might ground moral status, is mistaken. Our epistemic access to these ontological properties is indirect, by way of inferences to the best explanation for which I have shown that observable behaviour is not the only evidence available. When engaging in the potentially difficult enterprise of assessing robotic moral status, it is clearly epistemically most virtuous to employ all available evidence.

It has become clear that the core problem of ethical behaviourism is its behaviourism. That is, it fails due to its insistence that what goes on “on the inside” does not matter, and accordingly, its principled restriction of possible evidence for moral status to observable behavioural patterns. This was to be expected, since given the nature of the problem of moral status ascription, it is not at all obvious to adopt methodological behaviourism. As mentioned above, behaviourists like Skinner believed that mental states had no power to alter the causal chain from external stimuli to observable behaviour. So, if you think mental states do not matter in the explanation of behaviour, it makes sense to stay at the surface level of environmental stimuli and resulting manifest behaviour, trying to discover lawlike regularities. Hence, for Skinner, methodological behaviourism makes sense: inner mental states are irrelevant and**,** furthermore, not publicly observable, and therefore no proper object of study (Graham 2019).

On the contrary, for purposes of assessing moral status, methodological behaviourism doesn’t make sense. Moral status is intimately connected to the inner life of an entity like a robot: is it sentient, has it consciousness, is it intelligent, has it purposes of its own? Accordingly, inner ‘mental’ states are of utmost relevance to any plausible method for assessing robotic moral status. Contrary to ethical behaviourism’s slogan that “what goes on on the inside” does not matter, it matters everything.

From my critical discussion of ethical behaviourism, a few more constructive observations can be made. First, we cannot escape further developing our theories of what metaphysical properties ground moral status for robots (again, leaving out of consideration the views of Gunkel and Coeckelbergh). This, however, is a challenging task. Notoriously difficult concepts and phenomena such as intelligence, consciousness, and sentience, become even more puzzling when investigated in relation to robots (Cf. Schwitzgebel and Garza 2015). Consequently, robotic moral status is surrounded with significantly more moral uncertainty than human moral status.

For, secondly and somewhat surprisingly, in the case of humans EB does work: rough performative equivalence *is* sufficient to ascertain moral status. We need to see just as much human behaviour as is necessary to confirm that we are dealing with a fellow human. In the case of humans, there is no uncertainty with regard to its moral status, even if we lack agreement on a theoretical account of human moral status.

This is, thirdly, a reason to be careful with introducing androids that are difficult to distinguish from humans. For, inasmuch as we ascribe no or lesser moral status to androids, we would become uncertain with regard to the moral status of beings that look like they are human, but which might be an android as well.

The most important lesson, however, is that we should allow all evidence to contribute to determining a robot’s moral status.^18^

## Footnotes

1 See, for example, (Agar 2019; Bryson 2010, 2018; Coeckelbergh 2014; Gunkel 2018; Neely 2014; Nyholm 2020; Schwitzgebel and Garza 2015; Sparrow 2004).

2 In order to keep my discussion in this paper manageable, I do not consider alternative approaches to robotic moral status by Coeckelbergh (2010) and Gunkel (2018). Coeckelbergh and Gunkel argue that we should dispense with the approach of ascribing moral status based on metaphysical properties. Instead, inspired by Levinas and others, they argue for a relational approach: assessing, or perhaps rather, constructing a robot’s moral status can only be done in the process of a growing relationship with the robot, seeing its, or rather, her/his face.

3 All further references between brackets are to sections of Danaher’s paper.

4 As far as I can tell, Danaher’s argument for EB does not rely on this definition being unusually broad, and throughout the paper ‘behaviour’ seems to be used in its narrower everyday sense.

5 At still other places, Danaher calls rough performative equivalence to an entity having moral status “sufficient” evidence for moral status (sec. “Defending Premise (1)” and sec. “Different Ontologies objection”).

6 Danaher holds that today, cognitive (neuro)scientists are “still behaviouristic in their *methods*” (sec. “Defending Premise (1)”, Italics original) in the sense that they give primacy to externally observable behaviour. “They are just willing to hypothesize inner mental states to explain those external phenomena” (sec. “Defending Premise (1)”). However, as I just noted, their *un*willingness to do just that, is a defining characteristic of behaviourists. The epistemic importance of behaviour for cognitive science is something non-behaviourist are ready to acknowledge.

7 Of course, it is probably still be good *for us* to treat the robot dog with consideration, for reasons of protecting our character (Cf. Darling 2016).

8 Consider also: “Second, taking this theory [viz. EB, JS] onboard, the article asks the obvious question: What kind of performative threshold must robots cross in order to be afforded significant moral status? *Using analogies* with entities to whom we already afford significant moral status, it is argued that the performative threshold may be quite low and that robots may cross it soon (if not already).” (sec. “Introduction”, italics JS).

9 See (Avramides 2019) for discussion and distinguishing between epistemological and conceptual versions of the problem of other minds. I am concerned here with the epistemological problem of other minds.

10 Cf. (Sebo 2018), who speaks of the ‘moral problem of other minds’: when we are uncertain about whether other beings are sentient, how, morally speaking, ought we treat those beings?

11 Sentience is often mentioned as a property that grounds moral status. Cf. (Jaworska and Tannenbaum 2018; Sebo 2018).

12 Thanks to Wybo Houkes for putting it this way.

13 Note that this seems to render the whole Comparative Principle obsolete. For, it seems that in drawing the inferences from both animal and robotic behaviour to their respective moral statuses, we use our human perspective to state the hypothesis, e.g. that pain behaviour is to be explained by having mental pain states.

14 Cf. Chalmers (1996, p. 246) for this distinction.

15 It is also worth noting that the ascription of moral status to robots is theory-dependent in two distinct ways, each of which is a distinct source of uncertainty. First, we need agreement on a theory of which property or properties ground moral status. For a discussion of this problem, see (Coeckelbergh 2014). But then, second, for each of these properties we also have different philosophical theoretical accounts, that may differ in their view of how to ascertain the property in robots. Suppose that robots have (some) moral status if they can think. Followers of Searle (1980) will deny that a robotic AI could possibly think, while those of a more behaviourist inclination like Turing (1950), and also functionalists may be much more willing to grant such AI the capacity to think (Cf. Heil 2004, pp. 205–211). See for this second source of uncertainty also (Agar 2019).

16 Here is the original passage by Hauskeller: “Yet while it is quite possible that we are easily fooled, that our natural constitution as human (or more generally animal) beings makes it rather difficult for us not to ascribe self-awareness to a machine that behaves exactly as we would expect it to if it were really self-aware, as long as we have an alternative explanation for why it behaves that way (namely that it has been designed and programmed to do so) we have no good reason to believe that its actions are expressive of anything at all” (Hauskeller 2017, p. 207).

17 Interestingly, Sebo’s made-up numbers for purposes of his discussion differ only slightly, 12% vs. 8%. Moreover, he does not at all discuss the different efficient causes of both lobsters in respect to our estimation of the likelihood that they are sentient. My made-up estimated numbers would differ much more between the biological and the robot lobster. Assuming knowledge of the design process, I would assign the robot lobster a number in the order of magnitudes lower than the biological lobster.

18 For helpful feedback on earlier versions of this paper, I thank Sven Nyholm and my colleagues at our Eindhoven Philosophy and Ethics seminar. I also thank three anonymous reviewers from this journal for their helpful comments on an earlier draft of this paper.
